# Virtual Reality Functional Capacity Assessment Tool (VRFCAT-SL) in Parkinson’s Disease

**DOI:** 10.3233/JPD-212688

**Published:** 2021-10-12

**Authors:** Travis H. Turner, Alexandra Atkins, Richard S.E. Keefe

**Affiliations:** a Department of Neurology, Medical University of South Carolina, Charleston, SC, USA; b VeraSci, Inc., Durham, NC, USA; c Department of Psychiatry, Duke University Medical Center, Durham, NC, USA

**Keywords:** Assessment, cognitive capacity, functional capacity, functional outcome, mild cognitive impairment, neuropsychology, Parkinson’s disease, virtual reality

## Abstract

**Background:**

Cognitive impairment is common in Parkinson’s disease (PD) and highly associated with loss of independence, caregiver burden, and assisted living placement. The need for cognitive functional capacity tools validated for use in PD clinical and research applications has thus been emphasized in the literature. The Virtual Reality Functional Capacity Assessment Tool (VRFCAT-SL) is a tablet-based instrument that assesses proficiency for performing real world tasks in a highly realistic environment.

**Objective:**

The present study explored application of the VRFCAT-SL in clinical assessments of patients with PD. Specifically, we examined associations between VRFCAT-SL performance and measures of cognition, motor severity, and self-reported cognitive functioning.

**Methods:**

The VRFCAT-SL was completed by a sample of 29 PD patients seen in clinic for a comprehensive neuropsychological evaluation. Fifteen patients met Movement Disorders Society Task Force criteria for mild cognitive impairment (PD-MCI); no patients were diagnosed with dementia. Non-parametric correlations between VRFCAT-SL performance and standardized neuropsychological tests and clinical measures were examined.

**Results:**

VRFCAT-SL performance was moderately associated with global rank on neuropsychological testing and discriminated PD-MCI. Follow-up analyses found completion time was associated with visual memory, sustained attention, and set-switching, while errors were associated with psychomotor inhibition. No clinical or motor measures were associated with VRFCAT-SL performance. Self-report was not associated with VRFCAT-SL or neuropsychological test performance.

**Conclusion:**

The VRFCAT-SL appears to provide a useful measure of cognitive functional capacity that is not confounded by PD motor symptoms. Future studies will examine utility in PD dementia.

## INTRODUCTION

Parkinson’s disease (PD) is a progressive neurodegenerative disorder involving both motor and non-motor symptoms. Cognitive deficits are common in PD, with an approximate 40%meeting criteria for mild cognitive impairment (PD-MCI) and 80%or more progressing to Parkinson’s disease dementia (PDD) over the course of illness [1, 2]. Impaired cognition is highly associated with loss of independence and caregiver burden, and the strongest predictor of need for placement in assisted living facilities [3, 4].

In movement disorder clinics, neuropsychological evaluations are useful for determining whether subjective reporting of decline in memory and cognition by the patient is supported by objective findings of deficits on standardized tests [5]. Test performances determine the magnitude of impairment with cognitive domains, and the overall profile is interpreted with respect to differential diagnosis of changes typical for PD versus other etiologies. Neuropsychological tests also serve as a proxy for impairment in everyday life. For instance, if an individual performs poorly on standardized tests of attention, they may have difficulty performing tasks such as balancing a checkbook or counting pills [6]. Impairment on a test of verbal fluency may relate to word-finding difficulties in conversation [7]. However, such associations are certainly not perfect, and the need for ecologically valid measures of functional ability have been emphasized in the literature [8–10]. Such measures have also become increasingly emphasized by the Food and Drug Administration (FDA) as outcome measures in clinical trials [11].

Several informant-based questionnaires have been validated for use in assessing cognitive functional capacity in PD. However, knowledgeable informants are not always present during clinical evaluations, and these ratings can be biased by caregiver mood, burden, and other factors [12, 13]. Anosognosia of cognitive deficits [14, 15] and motor impairment [16] in PD also present limitations on the reliability of patient self-report.

Several standardized tests of real-world functioning have been developed and explored in PD populations. The Everyday cognition battery assesses medication use, financial planning, and food preparation and nutrition skills through a series of tasks that involve reading, episodic memory, working memory, and inductive reasoning [17]. It has been shown to be valid in assessing older adults with subjective cognitive decline and demonstrated sensitivity to treatment with rivastigmine in a clinical trial with PD [18]. However, administration procedures are rather complex and completion time of one hour limits application in both clinical evaluations and research. The University of California Performance-based Skills Assessment (UPSA) assesses abilities on tasks related to finances, communication, planning/organization, travel, and household chores and was developed to be used as a functional outcome measure in schizophrenia trials [19]. It takes about 30 minutes to administer and was recently validated in a PD population against a battery of standardized neuropsychological tests. Performance on the UPSA was found to be significantly related to standardized neuropsychological tests even after controlling for demographic factors and PD motor symptoms and discriminated PD with normal cognition from PD-MCI and PDD [20]. The Complex Task Performance Assessment (CTPA) requires examinees to execute the role of librarian in a structured format [21]. This includes managing inventory, listening and responding to telephone messages, and planning. It was developed for use in older adults and also explored in a recent study of PD patients without cognitive impairment. The investigators found that completion time discriminated PD patients from healthy controls, but error rates were similar [22]. Moreover, PD patients on average took about 40 minutes to complete the CTPA, which is near the published cutoff time.

The Virtual Reality Functional Capacity Assessment Tool (VRFCAT) is a tablet-based task that requires examinees to complete a series of real-world tasks in a virtual-reality environment [23]. The task was developed with grant support from the National Institute of Health (NIH) and the FDA to serve as a measure of functional improvement in clinical trials. Like the UPSA, it was originally standardized for use in schizophrenia, with subsequent studies demonstrating validity for assessment of functional capacity in older adults with cognitive impairment [24, 25]. The VRFCAT was developed to measure four different functional abilities: meal preparation, using transportation, shopping, and managing currency. These scenarios were developed using immersive “first-person” gaming technology. The realistic, interactive environment challenges the examinee to a series of tasks related to making a food dish. First, they explore a kitchen to see what items they have for the recipe. Then, they use a bus schedule to find a bus that will take them to a grocery store. They find and purchase the necessary items at the store and use the schedule once again to find the bus that will take them home. Exact change is needed for both bus trips and to purchase the food. Patients sequentially complete the scenarios through a progressive storyboard design. Across the 4 scenarios, there is total of 12 different tasks or “objectives”. For each objective, participants who are unable to complete the objective within a pre-specified time period are “pushed” to the next objective (referred to as “Forced Progression”). The assessment takes approximately 30 minutes to complete. The primary VRFCAT end point is Total Time. Secondary endpoints are Total Errors and Total Forced Progressions.

An abbreviated version of the test, the VRFCAT-SL was recently developed that reduces completion time to less than 15 minutes in healthy older adults. Aside from technical assistance that might be needed from the examiner, the task is self-administered and scoring is automated. Normative data with gender, age, and education correction are available from the publisher. These features, along with elimination of additional test materials and manipulatives, make the VRFCAT-SL particularly attractive for assessing functional abilities within the context of a comprehensive neuropsychological evaluation. There are also multiple versions available to reduce practice effect with serial testing.

We recently began administering the VRFCAT-SL to PD patients referred for clinical neuropsychological evaluations to assess functional capacity. The present study is a retrospective chart analysis to explore utility of the VRFCAT-SL as measure of functional capacity in clinical neuropsychological evaluations of subjective cognitive decline in PD. Specifically, we sought to determine whether VRFCAT-SL performance measures were associated with scores on standardized neuropsychological tests and sensitive to cognitive impairment in PD. We also wanted to assess the potential impact of motor disturbances on ability to complete the VRFCAT-SL. Finally, we examined whether the use of a standardized self-report scale for impact of cognition changes on instrumental ADLs, the Parkinson’s disease Cognitive Function Rating Scale (PD-CFRS) [26], would provide similar information regarding real-world functional ability.

## METHODS

This retrospective chart review was approved by the Human Subjects Protection Program at the Medical University of South Carolina, Pro00098834.

Participants were 30 consecutive patients followed in a tertiary care Movement Disorders clinic for idiopathic PD at an academic medical center who were referred for neuropsychological evaluation. PD diagnosis was made according to UK Brain Bank criteria by a fellowship-trained movement disorder neurologist. All patients were referred for neuropsychological evaluation for concerns of cognitive deficits as noted by the patient, informant, or referring neurologist. The VRFCAT-SL was administered to all patients seen during this time period (i.e., none were excluded from analysis). Administration was paused after these 30 assessments to perform these analyses related to quality assurance. One participant was unable to complete the VRFCAT-SL due to visual impairment (advanced age-related macular degeneration); this individual was also not able to complete other tests with small visual stimuli, the PD-CFRS, and other self-report inventories.

The first author (THT) performed all neuropsychological evaluations and administered the VRFCAT-SL. A core battery of tests was administered that included: Mattis Dementia Rating Scale (DRS), Brief Visuospatial Memory Test [27] (BVMT-R, Total Learning and Delayed Recall), Hopkins Verbal Learning Test [28] (HVLT-R, Total Learning and Delayed Recall), Judgment of Line Orientation (JOLO) [29], Neuropsychological Assessment Battery (NAB) [30]: Numbers & Letters A (Time and Errors), Digits Forward and Digits Backward, and Naming subtest; Hayling Sentence Completion Test [31] (Direct Time, Inhibition Time, Inhibition Errors), Controlled Oral Word Association Test (FAS and Animals) and Trail Making Test (Trails A, Trails B) from Halstead-Reitan Battery [32]. Other tests were administered as indicated. The Geriatric Depression Scale (GDS) [33] and Geriatric Anxiety Inventory (GAI) [34] were also administered. Raw scores were corrected for demographics according to test manuals (BVMT, HVLT, and Hayling corrected for age; NAB corrected for age and education; COWAT and Trails for age, gender, education, and ethnicity) and converted to scaled scores. Diagnosis of cognitive status was based on clinical history and performance on standardized neuropsychological testing in keeping with Movement Disorders Society Task Force Level II criteria for PD-MCI [35]. Specifically, the presence of two or more scores within one domain falling more than 1.5 SD below normative expectations were interpreted to reflect MCI.

All standardized testing was completed first, followed by the PD-CFRS, and then the VRFCAT-SL. Performance on the VRFCAT-SL was interpreted qualitatively with respect to implications for functional independence but not was not considered for diagnosis. For all analyses, VRFCAT-SL performances were corrected for age, gender, and education using normative data from the test publisher. For a subset of the sample (*n* = 20), the Movement Disorders Society’s version of the Unified Parkinson’s disease rating scale (MDS-UPDRS) [36] Part III ratings made by the referring movement disorder neurologist within 3 months of assessment while subjectively “on” medication were available.

Measures of central tendency were examined for VRFCAT-SL outcomes. Corrected scores were strongly skewed (Time = –0.968, Errors = –1.564, Forced Progressions = –2.921) and did not meet assumptions of normal distribution (see Supplementary Figure 1). Notably, only 4 patients had any forced progressions, and just one patient had 2 forced progressions. Accordingly, non-parametric tests were used for all analyses (i.e., Spearman-Brown for correlations and Mann-Whitney U-tests for group comparisons).

To examine association between VRFCAT-SL performance and overall cognition, a global cognitive index for each participant was derived based on the average rank (higher = better) for all standardized neuropsychological tests excluding the DRS-2. Similarly, a global score on the VRFCAT-SL was derived based on the average rank for its primary outcome measures (time, errors, and forced progressions). In exploring associations between VRFCAT-SL measures and neuropsychological test performances, the Bonferroni method was used to control for increased Type-I error rate associated with multiple comparisons (i.e., 17 measures, *p* < 0.002). SPSS version 25 ® was used for all statistical analyses.

## RESULTS

Measures of central tendency for demographic, clinical characteristics, and test performances are provided for the entire sample, and separately by cognitive status group in Table 1. Boxplots illustrating ranges for VRFCAT-SL outcome measures relative to standardized neuropsychological test performances are provided in Supplementary Figure 2. For the overall sample, the average completion time for the VRFCAT-SL was 791 seconds (SD = 242) or about 13 minutes, with a minimum completion time of 504 seconds, and maximum completion time of 1461 seconds.

**Table 1:** Means (SD) for demographics, clinical characteristics, and test performances of the sample. *P*-values for comparisons between cognitive status groups are derived from *t*-tests except for VRFCAT-SL measures which reflect Mann-Whitney U-tests

	Measure	Entire Sample (*n* = 29)	PD Normal (*n* = 14)	PD-MCI (*n* = 15)	group difference
Demographic	Gender (Female)	12 (41%)	5 (36%)	7 (47%)	n.s.
	Age	66.62 (7.64)	65. 50 (7.38)	67.67 (8.04)	n.s.
	Education	15.66 (2.38)	16.07 (2.12)	15.27 (2.60)	n.s.
Clinical	Duration of Illness	8.66 (4.79)	9.14 (5.11)	8.20 (4.60)	n.s.
	HY Stage	2.38 (0.53)	2.18 (0.58)	2.57 (0.42)	*p* = 0.050
	LEDD	980 (471)	1091 (576)	877 (335)	n.s.
	MDS-UPDRS Part III	29.45 (11.38)	29.30 (14.03)	29.60 (8.73)	n.s.
Cognitive (T)	Mattis DRS-2 Total Score	139.95 (3.56)	141.11 (2.08)	139.00 (4.29)	n.s.
	BVMT Total Learning	45.78 (11.77)	51 (9.35)	40.93 (11.98)	*p* = 0.022
	BVMT Delayed Recall	46.89 (12.66)	53.85 (9.51)	40.43 (11.98)	*p* = 0.003
	HVLT-R Total Learning	45.08 (9.96)	48.42 (8.78)	42.21 (10.32)	n.s.
	HVLT-R Delayed Recall	44.58 (9.85)	49.42 (8.74)	40.43 (9.04)	*p* = 0.017
	NAB Digits Fwd	48.48 (9.95)	52.23 (10.00)	45 (8.86)	n.s.
	NAB Digits Bkwd	49.96 (7.09)	52.77 (6.82)	47.36 (6.51)	*p* = 0.045
	NAB Numbers &Letters A Time	47.45 (13.66)	52.3 (13.68)	42.6 (12.42)	n.s.
	NAB Numbers &Letters A Errors	46.5 (10.54)	52 (7.41)	41 (10.6)	*p* = 0.015
	NAB Naming	55.92 (2.24)	55.5 (2.39)	56.33 (2.1)	n.s.
	NAB Figure Copy	56.86 (7.45)	58.45 (5.70)	55.27 (8.86)	n.s.
	FAS	50.85 (10.98)	54.69 (8.83)	47.29 (11.86)	n.s.
	Animals	52.04 (12.61)	58.23 (10.72)	46.29 (11.74)	*p* = 0.011
	Trails A	49.93 (9.82)	53.62 (9.08)	46.5 (9.53)	n.s.
	Trails B	49.37 (14.66)	57 (10.21)	42.29 (14.87)	*p* = 0.006
	Hayling Sentence Inhibition Errors	48.67 (8.03)	49.61 (6.54)	47.64 (9.62)	n.s.
VRFCAT-SL (raw)	Completion Time (seconds)	791 (242)	637 (81)	935 (255)
	Errors	2.41 (2.23)	1.21 (1.18)	3.53 (2.42)
	Forced Progressions	0.17 (0.468)	0 (0)	0.33 (0.62)
VRFCAT-SL (T)	Completion Time	48.60 (13.49)	57.13 (3.85)	40.88 (14.04)	*p* = 0.001
	Errors	47.90 (17.75)	56.13 (7.96)	42.67 (16.18)	*p* = 0.005
	Forced Progressions	49.61 (11.65)	53.63 (1.09)	46.87 (12.78)	n.s.
Self-report	CFRS	4.46 (3.96)	4.0 (3.63)	4.85 (4.32)	n.s.
	GDS	8.30 (7.28)	9.46 (9.40)	7.21 (4.74)	n.s.
	GAI	3.52 (3.98)	4.38 (4.81)	2.71 (3.00)	n.s.

### Association with standardized neuropsychological tests

**Fig. 1:**
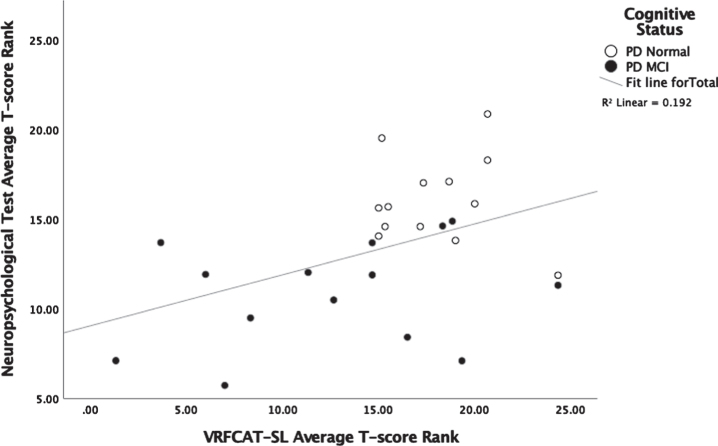
Association between VRFCAT-SL performance and standardized neuropsychological test performance.

Spearman-brown correlation analysis assessed relationship between overall VRFCAT performance measure (i.e., corrected Time, Errors, and Forced Progressions) and standardized neuropsychological testing. As shown in Fig. 1, there was a modest association between overall ranks, *rho* = 0.404, *p* = 0.037. The relationship was not driven by outliers. Overall standardized neuropsychological test performance was associated VRFCAT-SL time, *rho* = 0.532, *p* = 0.004, but not Errors or Forced Progressions.

Relationships between VRFCAT-SL outcome measures and primary neuropsychological outcome measures were also explored. As shown in Table 2, T-score for VRFCAT-SL Time was positively associated with T-scores for NAB Numbers & Letters A Time, *rho* = 0.457, *p* = 0.043, Trails B Time, *rho* = 0.472, *p* = 0.013, and BVMT-R Delayed Recall, *rho* = 0.444, *p* = 0.02. The T-Score for VRFCAT-SL Errors was inversely correlated with Inhibition Errors (raw) on the Hayling, *rho* = –0.561, *p* = 0.005, indicating greater errors on the VRFCAT-SL were associated with increased errors on the Haylling. T-score for VRFCAT-SL forced progressions was positively correlated with T-score for NAB Naming, *rho* = 0.499, *p* = 0.013. All correlations were thus in the expected direction that improved VRFCAT-SL performance was associated with improved neuropsychological test performance. No other correlations were significant at the *p* < 0.05 level, and none of the associations exceeded Bonferroni correction for multiple comparison (i.e., 17 measures, *p* < 0.002). Raw total score from the DRS-2 was not significantly associated with any VRFCAT-SL measure.

**Table 2:** Spearman-Brown correlations (*p* value, n) between demographically-corrected scores on standardized neuropsychological tests and VRFCAT-SL measures

	VRFCAT-SL Time	VRFCAT-SL Errors	VRFCAT-SL Forced Progressions
BVMT Total Learning	0.345	0.152	–0.095
	0.078	0.449	0.637
	27	27	27
BVMT Delayed Recall	0.444^*^	0.135	–0.105
	**0.02**	0.503	0.602
	**27**	27	27
HVLT-R Total Learning	0.26	0.258	0.143
	0.199	0.203	0.485
	26	26	26
HVLT-R Delayed Recall	0.206	0.195	–0.115
	0.314	0.339	0.575
	26	26	26
NAB Digits Fwd	0.192	0.266	0.047
	0.339	0.179	0.816
	27	27	27
NAB Digits Bkwd	0.237	–0.169	0.178
	0.235	0.4	0.375
	27	27	27
NAB Numbers &Letters A Time	0.457^*^	–0.001	0.264
	**0.043**	0.997	0.26
	**20**	20	20
NAB Numbers &Letters A Errors	0.279	0.151	0.013
	0.233	0.524	0.957
	20	20	20
NAB Naming	–0.06	0.201	0.499^*^
	0.781	0.346	**0.013**
	24	24	**24**
NAB Figure Copy	–0.224	0.115	–0.019
	0.316	0.611	0.933
	22	22	22
FAS	0.245	–0.133	0.218
	0.219	0.508	0.274
	27	27	27
Animals	0.224	0.321	0.072
	0.26	0.103	0.722
	27	27	27
Trails A	0.325	–0.26	0.09
	0.098	0.19	0.654
	27	27	27
Trails B	0.472^*^	–0.23	0.063
	**0.013**	0.249	0.755
	**27**	27	27
Hayling Initiation	–0.202	–0.168	0.109
	0.356	0.443	0.62
	23	23	23
Hayling Inhibition Time	–0.026	–0.158	0.181
	0.907	0.473	0.407
	23	23	23
Hayling Inhibition Errors	0.096	0.561^ **^	–0.09
	0.663	0.005	0.683
	23	23	23

### Sensitivity to cognitive status

About one-half of the sample (*n* = 15) met MDS Task Force Level II criteria for PD-MCI. Independent sample Mann-Whitney U-tests compared performance on VRFCAT-SL outcomes between cognitive status groups. Statistically significant group differences were found for Time, *p* = 0.001, and Errors, *p* = 0.005. Statistically significant group differences were not observed for Forced Progressions; however, all 4 patients with a Forced Progression were in the PD-MCI group. Notably, group differences based on cognitive status were also not observed on the DRS-2, *p* = 0.331.

### Association with motor symptoms

Spearman-Brown correlation analyses explored impact of PD motor disturbance on VRFCAT-SL performance. No statistically significant correlations were observed between any VRFCAT-SL measure and duration of illness (years), levodopa equivalent daily dose, or MDS-UPDRS Part III scores. When analyzed separately by cognitive status, a trend was seen for the relationship between Errors and durations of illness, *rho* = 0.500, *p* = 0.069 in the cognitively normal group (*n* = 14). In the PD-MCI group (*n* = 15), a statistically significant correlation was observed between Time and LEDD, *rho* = –0.588, *p* = 0.021, and a trend was observed between Errors and LEDD, *rho* = –0.477, *p* = 0.072.

### Association with self-reported impact of cognition on instrumental ADLs

The composite rank for VRFCAT-SL performance was not associated with self-report on the PD-CFRS, *rho* = 0.067, *p* = 0.774. Follow-up analyses did not reveal any statistically significant associations with VRFCAT-SL Time, Errors, or Forced Progressions. Self-report on the PD-CFRS was also not associated with composite rank on the standardized neuropsychological tests, *rho* = –0.289, *p* = 0.170. Though falling short of a trend, the direction of this association suggests greater subjective impairment in those with worse performance on neuropsychological testing. Mann-Whitney U-tests indicated that scores from the CFRS also did not differ between cognitive status groups, *p* = 0.531. PD-CFRS scores were also not associated with motor disturbance (MDS-UPDRS Part III scores), *rho* = 0.199, *p* = 0.428. However, clinically significant associations were observed between PD-CFRS ratings and self-report depression (GDS), *rho* = 0.621, *p* = 0.001, and anxiety (GAI), *rho* = 0.412, *p* = 0.046.

## DISCUSSION

Current results support use of the VRFCAT-SL for providing an efficient global assessment of functional cognition in PD patients with subjective cognitive decline. A modest correlation was observed between composite ranks for overall VRFCAT-SL and standardized neuropsychological measures. VRFCAT-SL performance measures also discriminated patients meeting MDS Task Force Level II criteria for PD-MCI from PD patients with normal cognition. Importantly, there were no indications that motor impairment significantly impacts task performance. Taken together, our findings using the VRFCAT-SL are consistent with those previously reported using other functional capacity tools. As with the CTPA, the VRFCAT-SL identified mild cognitive impairment in PD [22]. This study also replicated findings using the UPSA, which not only showed that performances differed between PD normal and PD-MCI, but those performances were associated with cognition independent of motor symptoms [21]. However, the average completion time of about 13 minutes for the VRFCAT-SL was found to be much lower than reported times for the UPSA (30 minutes), CTPA (40 minutes), and ECB (60 minutes) [17]. Thus, in addition to tablet-based administration and automated scoring, reduced time demands associated with the VRFCAT-SL facilitates inclusion in more comprehensive clinical and research batteries.

Findings from this study did not support the use of patient’s self-report on the PD-CFRS for assessing impact of cognitive deficits on instrumental ADLs. While Kulisevsky et al. suggest use of the PD-CFRS for self-report [26], in our sample PD-CFRS ratings were not associated with VRFCAT-SL performance or standardized neuropsychological measures and did not differ between cognitive diagnosis groups. Rather than reflecting cognition and motor function, PD-CFRS ratings in this sample appeared to capture anxiety and depressive mood symptomatology. This finding is in keeping with a number of prior studies describing anosognosia for both cognitive and motor disturbances in PD [14–16].

One limitation of the VRFCAT-SL with respect to providing a comprehensive functional capacity index in PD is the lack of a medication management task. Most patients with progressed PD follow a complex regimen involving variable doses of one or more dopamine replacement therapies throughout the day, as well as medications that might be taken for non-motor symptoms and other health conditions. The ability to reliably take medications as prescribed has presents a significant cognitive challenge and obvious relevance to overall functioning. Lack of a medication condition is also a weakness of the UPSA and CTPA. The ECB includes several items that require participants to remember information contained on a pill bottle and answer multiple choice questions but does not measure ability to actually follow a medication schedule. As such, this would appear to be more of a test of attention, learning, and memory with medication-themed stimuli, rather than a medication adherence task, per se. The Medication Management Abilities Assessment (MMAA) [37] was specifically designed to assess to evaluate this domain. The MMAA requires examinees to learn instructions for taking 4 different medications, retain this information over a one-hour delay, and properly distribute the correct number of pills along with instructions (e.g., with or without food) to the rater. In a study by Pirogovsky et al., PD patients meeting criteria for mild cognitive impairment based on standardized neuropsychological evaluation performed worse than those with normal cognition and a healthy control group [10]. The ecological validity of the VRFCAT-SL in PD might therefore be improved if medication adherence were assessed, perhaps by requiring examinees to observe the passing of time and take virtual pills as directed within a certain time window on several occasions throughout the task.

A methodological limitation of this study is that the provider (THT) performed neuropsychological testing, administered the VRFCAT-SL, and interpreted test performances. While cognitive status was based on clinical history and standardized neuropsychological testing, the possibility of diagnostic bias from observing VRFCAT-SL performance must be considered with respect to sensitivity and specificity estimates for discriminating PD-MCI. This study also included a relatively small sample of PD patients, and only a subset of participants had MDS-UDPRS Part III scores thus limiting generalizability. Future studies with neuropsychological evaluation independent of VRFCAT-SL administration are therefore recommended.

In the course of this study, no patients were found to meet MDS Task Force criteria for dementia in PD. This is not particularly surprising given that referring Movement Disorder neurologists generally request neuropsychological testing to identify mild cognitive impairment, while diagnosis of dementia is based on more obvious deficits and loss of independence. Future studies are thus needed to determine whether this can be used effectively in PD dementia populations.

Taken together, the results from this retrospective chart review provide provisional support for the VRFCAT-SL as an efficient tool for obtaining a global estimate of cognitive functioning in moderately advanced PD. Compared to other standardized functional capacity tools that have been used in PD, the tablet-based VRFCAT-SL eliminates the need for manipulatives, reduces administration and scoring complexities, and appears to be less burdensome with respect to time. Further investigation is required to evaluate test-retest reliability and determine whether it provides an objective measure of functional cognitive capacity across the full spectrum of disease progression.

## Supplementary Material

Supplementary Material

## References

[1] Aarsland D , Andersen K , Larsen JP , Lolk A , Kragh-Sorensen P (2003) Prevalence and characteristics of dementia in Parkinson disease: an 8-year prospective study. Arch Neurol 60, 387–392. 12633150 10.1001/archneur.60.3.387

[2] Baiano C , Barone P , Trojano L , Santangelo G (2020) Prevalence and clinical aspects of mild cognitive impairment in Parkinson’s disease: A meta-analysis. Mov Disord 35, 45–54. 31743500 10.1002/mds.27902

[3] Barone P , Antonini A , Colosimo C , Marconi R , Morgante L , Avarello TP , Bottacchi E , Cannas A , Ceravolo G , Ceravolo R , Cicarelli G , Gaglio RM , Giglia RM , Iemolo F , Manfredi M , Meco G , Nicoletti A , Pederzoli M , Petrone A , Pisani A , Pontieri FE , Quatrale R , Ramat S , Scala R , Volpe G , Zappulla S , Bentivoglio AR , Stocchi F , Trianni G , Dotto PD ; PRIAMO study group (2009) The PRIAMO study: A multicenter assessment of nonmotor symptoms and their impact on quality of life in Parkinson’s disease. Mov Disord 24, 1641–1649. 19514014 10.1002/mds.22643

[4] Weintraub D , Burn DJ (2011) Parkinson’s disease: the quintessential neuropsychiatric disorder. Mov Disord 26, 1022–1031. 21626547 10.1002/mds.23664 PMC3513835

[5] Marras C , Tröster AI , Kulisevsky J , Stebbins GT (2014) The tools of the trade: a state of the art “How to Assess Cognition” in the patient with Parkinson’s disease. Mov Disord 29, 584–596. 24757108 10.1002/mds.25874

[6] Manning KJ , Clarke C , Lorry A , Weintraub D , Wilkinson JR , Duda JE , Moberg PJ (2012) Medication management and neuropsychological performance in Parkinson’s disease. Clin Neuropsychol 26, 45–58. 22150514 10.1080/13854046.2011.639312 PMC3646561

[7] Matison R , Mayeux R , Rosen J , Fahn S (1982) “Tip-of-the-tongue” phenomenon in Parkinson disease. Neurology 32, 567–570. 7200216 10.1212/wnl.32.5.567

[8] Cahn DA , Sullivan EV , Shear PK , Pfefferbaum A , Heit G , Silverberg G (1998) Differential contributions of cognitive and motor component processes to physical and instrumental activities of daily living in Parkinson’s disease. Arch Clin Neuropsychol 13, 575–583. 14590618

[9] Young TL , Granic A , Yu Chen T , Haley CB , Edwards JD (2010) Everyday reasoning abilities in persons with Parkinson’s disease. Mov Disord 25, 2756–2761. 20939079 10.1002/mds.23379 PMC3003758

[10] Pirogovsky E , Schiehser DM , Obtera KM , Burke MM , Lessig SL , Song DD , Litvan I , Filoteo JV (2014) Instrumental activities of daily living are impaired in Parkinson’s disease patients with mild cognitive impairment. Neuropsychology 28, 229–237. 24417192 10.1037/neu0000045

[11] U.S. Food and Drug Administration (FDA), Center for Drug Evaluation and Research (CDER) (2013) Guidance for Industry: Alzheimer’s Disease: Developing Drugs for the Treatment of Early Stage Disease. Department of Health and Human Services, Washington, DC: U.S.

[12] Argüelles S , Loewenstein DA , Eisdorfer C , Argüelles T (2001) Caregivers’ judgments of the functional abilities of the Alzheimer’s disease patient: impact of caregivers’ depression and perceived burden. J Geriatr Psychiatry Neurol 14, 91–98. 11419574 10.1177/089198870101400209

[13] Loewenstein DA , Argüelles S , Bravo M , Freeman RQ , Argüelles T , Acevedo A , Eisdorfer C (2001) Caregivers’ judgments of the functional abilities of the Alzheimer’s disease patient: a comparison of proxy reports and objective measures. J Gerontol B Psychol Sci Soc Sci 56, 78–84. 10.1093/geronb/56.2.p78 11245361

[14] Pillai JA , Bonner-Jackson A , Floden D , Fernandez H , Leverenz JB (2018) Lack of accurate self-appraisal is equally likely in MCI from Parkinson’s disease and Alzheimer’s disease. Mov Disord Clin Pract 5, 283–289. 30363404 10.1002/mdc3.12606 PMC6174380

[15] Orfei MD , Assogna F , Pellicano C , Pontieri FE , Caltagirone C , Pierantozzi M , Stefani A , Spalletta G (2018) Anosognosia for cognitive and behavioral symptoms in Parkinson’s disease with mild dementia and mild cognitive impairment: Frequency and neuropsychological/neuropsychiatric correlates. Parkinsonism Relat Disord 54, 62–67. 29709507 10.1016/j.parkreldis.2018.04.015

[16] Maier F , Prigatano GP (2017) Impaired self-awareness of motor disturbances in Parkinson’s disease. Arch Clin Neuropsychol 32, 802–809. 29028874 10.1093/arclin/acx094

[17] Allaire JC , Marsiske M (1999) Everyday cognition: age and intellectual ability correlates. Psychol Aging 14, 627–644. 10632150 10.1037//0882-7974.14.4.627 PMC2904910

[18] Mamikonyan E , Xie SX , Melvin E , Weintraub D (2015) Rivastigmine for mild cognitive impairment in Parkinson disease: a placebo-controlled study. Mov Disord 30, 912–918. 25914281 10.1002/mds.26236

[19] Patterson TL , Goldman S , McKibbin CL , Hughs T , Jeste DV (2001) UCSD Performance-Based Skills Assessment: development of a new measure of everyday functioning for severely mentally ill adults. Schizophr Bull 27, 235–245. 11354591 10.1093/oxfordjournals.schbul.a006870

[20] Holden SK , Medina LD , Hoyt B , Sillau SH , Berman BD , Goldman JG , Weintraub D , Kluger BM (2018) Validation of a performance-based assessment of cognitive functional ability in Parkinson’s disease. Mov Disord 33, 1760–1768. 30306618 10.1002/mds.27487 PMC6261681

[21] Wolf TJ , Morrison T , Matheson L (2008) Initial development of a work-related assessment of dysexecutive syndrome: the Complex Task Performance Assessment. Work 31, 221–228. 18957739

[22] Davis A , Wolf TJ , Foster ER (2019) Complex Task Performance Assessment (CTPA) and functional cognition in people with Parkinson’s disease. Am J Occup Ther 73, 1–9. 10.5014/ajot.2019.031492 PMC6813512 31484030

[23] Keefe RSE , Davis VG , Atkins A , Vaughn A , Patterson T , Narasimhan M , Harvey PD (2016) Validation of a computerized test of functional capacity. Schizophr Res 175, 90–96. 27091656 10.1016/j.schres.2016.03.038 PMC4958510

[24] Atkins AS , Stroescu I , Spagnola NB , Davis VG , Patterson TD , Narasimhan M , Harvey PD , Keefe RS (2015) Assessment of age-related differences in functional capacity using the Virtual Reality Functional Capacity Assessment Tool (VRFCAT). J Prev Alzheimers Dis 2, 121–127. 26618145 10.14283/jpad.2015.61 PMC4657736

[25] Atkins AS , Khan A , Ulshen D , Vaughan A , Balentin D , Dickerson H , Liharska LE , Plassman B , Welsh-Bohmer K , Keefe RSE (2018) Assessment of instrumental activities of daily living in older adults with subjective cognitive decline using the Virtual Reality Functional Capacity Assessment Tool (VRFCAT). J Prev Alzheimers Dis 5, 216–234.3029817910.14283/jpad.2018.28

[26] Kulisevsky J , Fernández de Bobadilla R , Pagonabarraga J , Martínez-Horta S , Campolongo A , García-Sánchez C , Pascual-Sedano B , Ribosa-Nogué R , Villa-Bonomo C (2013) Measuring functional impact of cognitive impairment: validation of the Parkinson’s disease cognitive functional rating scale. Parkinsonism Relat Disord 19, 812–817. 23773412 10.1016/j.parkreldis.2013.05.007

[27] Benedict RHB (1997) Brief visuospatial memory test Revised professional manual. Psychological Assessment Resources, Inc, Odessa, FL.

[28] Benedict RHB , Schretlen D , Groninger L , Brandt J (1998) Hopkins Verbal Learning Test - Revised: normative data and analysis of inter-form and test-retest reliability. Clin Neuropsychol 12, 43–55.

[29] Benton AL , Hamsher K , Varney N , Spreen O (1978) Visuospatial judgment: A clinical test. Arch Neurol 35, 364–367. 655909 10.1001/archneur.1978.00500300038006

[30] White T , Stern R (2003) Neuropsychological assessment battery. Psychological Assessment Resources, Lutz, Florida.

[31] Burgess PW , Shallice T (1996) Response suppression, initiation, and strategy using following frontal lobe lesions. Neuropsychologia 34, 263–272. 8657357 10.1016/0028-3932(95)00104-2

[32] Heaton RK , Miller SW , Taylor MJ , Grant I , PAR Staff (2005) Revised comprehensive norms for an expanded Halstead-Reitan Battery: Demographically adjusted neuropychological norms for African American and Caucasian adults scoring program. Psychological Assessment Resources, Lutz, FL.

[33] Yesavage JA , Brink TL , Rose TL , Lum O , Huang V , Adey M , Leirer VO (1982) Development and validation of a geriatric depression screening scale: a preliminary report. J Psychiatr Res 17, 37–49. 7183759 10.1016/0022-3956(82)90033-4

[34] Pachana NA , Byrne GJ , Siddle H , Koloski N , Harley E , Arnold E (2007) Development and validation of the Geriatric Anxiety Inventory. Int Psychogeriatr 19, 103–114. 16805925 10.1017/S1041610206003504

[35] Litvan I , Aarsland D , Adler CH , Goldman JG , Kulisevsky J , Mollenhauer B , Rodriguez-Oroz MC , Tröster AI , Weintraub D (2011) MDS Task Force on mild cognitive impairment in Parkinson’s disease: critical review of PD-MCI. Mov Disord 26, 1814–1824. 21661055 10.1002/mds.23823 PMC3181006

[36] Goetz CG , Fahn S , Martinez-Martin P , Poewe W , Sampaio C , Stebbins GT , Stern MB , Tilley BC , Dodel R , Dubois B , Holloway R , Jankovic J , Kulisevsky J , Lang AE , Lees A , Leurgans S , LeWitt PA , Nyenhuis D , Olanow CW , Rascol O , Schrag A , Teresi JA , Van Hilten JJ , LaPelle N (2007) Movement Disorder Society-sponsored revision of the Unified Parkinson’s Disease Rating Scale (MDS-UPDRS): Process, format, and clinimetric testing plan. Mov Disord 22, 41–47. 17115387 10.1002/mds.21198

[37] Patterson TL , Lacro J , McKibbin CL , Moscona S , Hughs T , Jeste DV (2002) Medication management ability assessment: results from a performance-based measure in older outpatients with schizophrenia. J Clin Psychopharmacol 22, 11–19. 11799337 10.1097/00004714-200202000-00003

