# Supplementary material for: Virtual Reality Functional Capacity Assessment Tool (VRFCAT-SL) in Parkinson’s Disease
Source: J Parkinsons Dis. 2021 Oct 12;11(4):1917–25. doi: 10.3233/JPD-212688 (PMC8609696; doi:10.3233/JPD-212688)

Supplementary Material

Virtual Reality Functional Capacity Assessment Tool (VRFCAT-SL) in Parkinson’s Disease

Supplementary Figure 1. Distributions of VRFCAT-SL performance outcomes (age, gender, and education corrected T-scores).

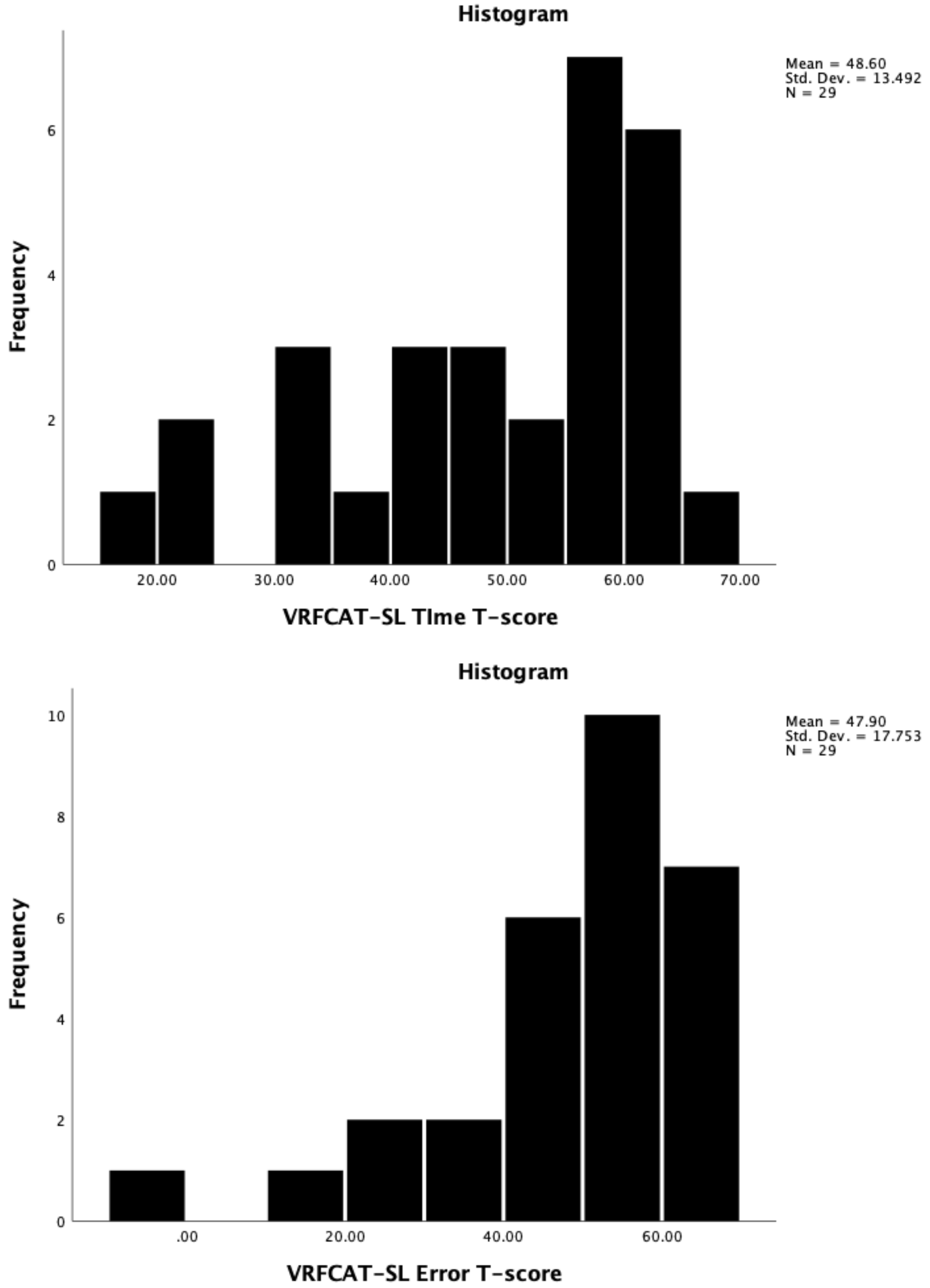

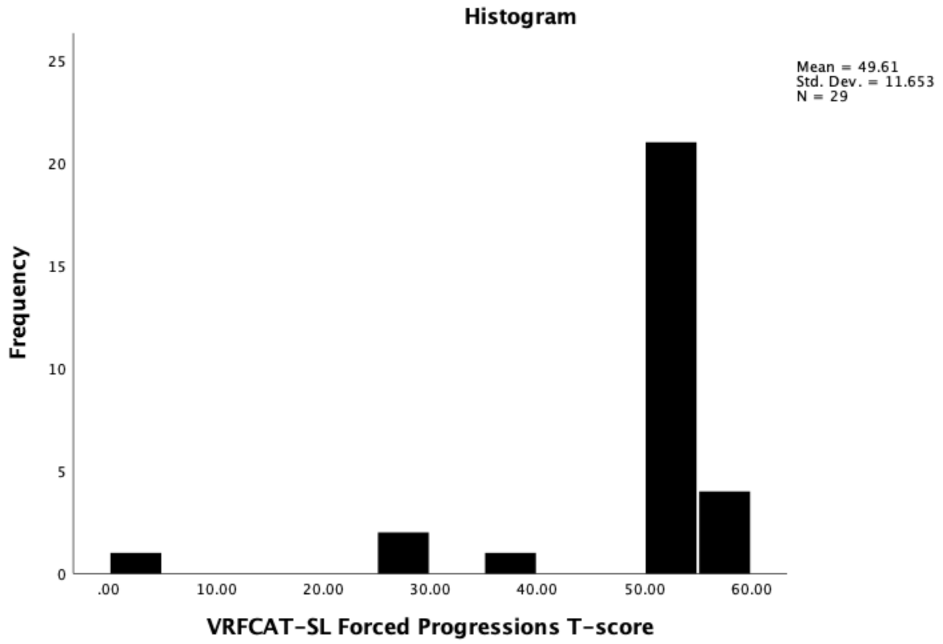
Histogram
Mean = 49.61
Std. Dev. = 11.653
N = 29
Frequency
25
20
15
10
5
0
.00
10.00
20.00
30.00
40.00
50.00
60.00
VRFCAT-SL Forced Progressions T-score

Supplementary Figure 2. Boxplots showing distributions of T-scores for VRFCAT-SL measures relative to standardized neuropsychological tests.

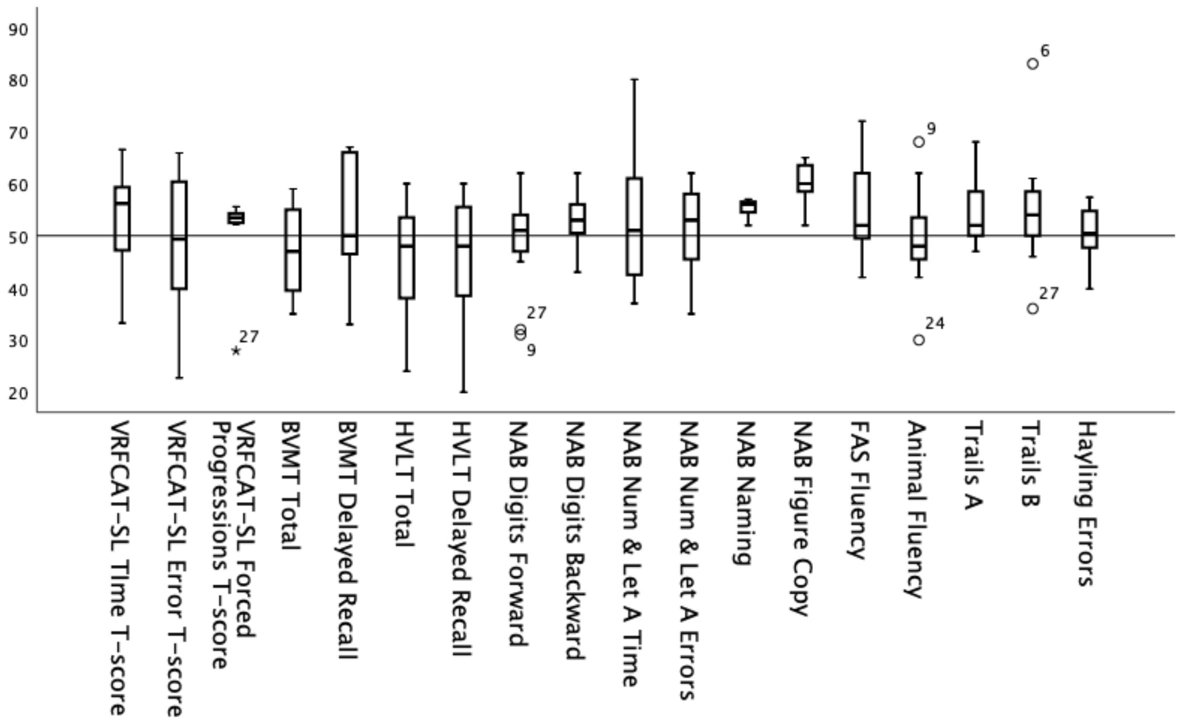

Supplement: Supplementary Material [file jpd-11-jpd212688-s001.pdf]
